# Microbial Diversity in Deep-sea Methane Seep Sediments Presented by SSU rRNA Gene Tag Sequencing

**DOI:** 10.1264/jsme2.ME12032

**Published:** 2012-04-18

**Authors:** Takuro Nunoura, Yoshihiro Takaki, Hiromi Kazama, Miho Hirai, Juichiro Ashi, Hiroyuki Imachi, Ken Takai

**Affiliations:** 1 Subsurface Geobiology & Advanced Research (SUGAR) Project, Extremobiosphere Research Program, Institute of Biogeosciences, Japan Agency for Marine-Earth Science & Technology (JAMSTEC), 2–15 Natsushima-cho, Yokosuka 237–0061, Japan; 2 Department of Ocean Floor Geoscience, Atmosphere and Ocean Research Institute, The University of Tokyo, 5–1–5, Kashiwanoha, Kashiwa, Chiba 277–8564, Japan

**Keywords:** methane seep, sediment, deep-sea, tag-sequencing

## Abstract

Microbial community structures in methane seep sediments in the Nankai Trough were analyzed by tag-sequencing analysis for the small subunit (SSU) rRNA gene using a newly developed primer set. The dominant members of *Archaea* were Deep-sea Hydrothermal Vent Euryarchaeotic Group 6 (DHVEG 6), Marine Group I (MGI) and Deep Sea Archaeal Group (DSAG), and those in *Bacteria* were *Alpha-*, *Gamma-*, *Delta-* and *Epsilonproteobacteria*, *Chloroflexi*, *Bacteroidetes*, *Planctomycetes* and *Acidobacteria*. Diversity and richness were examined by 8,709 and 7,690 tag-sequences from sediments at 5 and 25 cm below the seafloor (cmbsf), respectively. The estimated diversity and richness in the methane seep sediment are as high as those in soil and deep-sea hydrothermal environments, although the tag-sequences obtained in this study were not sufficient to show whole microbial diversity in this analysis. We also compared the diversity and richness of each taxon/division between the sediments from the two depths, and found that the diversity and richness of some taxa/divisions varied significantly along with the depth.

In deep-sea anoxic methane seep sediments, methanotrophic *Euryarchaeota* and syntrophic sulfate-reducing *Deltaproteobacteria* oxidize methane anaerobically with the following equation:

(4) $${\text{CH}}_{4}+{{\text{SO}}_{4}}^{2-}\to {\text{HCO}}^{3-}+{\text{HS}}^{-}+{\text{H}}_{2}\text{O}$$

and this syntrophic reaction is one of the most abundant processes of methane seep ecosystems (16). In contrast to the subsurface sediments, sulfur oxidizers belong to *Gammaproteobacteria* and *Epsilonproteobacteria* that utilize the high sulfide fluxes derived from anaerobic oxidation of methane (AOM) (14, 16, 20, 31), aerobic methanotroph in *Gammaproteobacteria* (15, 40), and ammonia-oxidizing marine group I (MGI) *Archaea* ‘*Nitrosopumilales*’ (3, 9, 11, 14) distributed on the seafloor. Therefore, the ecosystem is regarded as a chemosynthetic oasis in the deep-sea floor environment that is supported by these primary producers in comparison to the ambient seafloor sediment ecosystem that is restricted by the input of fresh detritus (16, 34). In addition, other uncultivated lineages of *Archaea* such as the Deep-sea Archaeal Group (DSAG, also known as Marine Benthic Group B), Marine Benthic Group D (MBGD) and Miscellaneous Crenarchaeotic Group (MCG, also known as Marine Benthic Group C) and diverse bacterial groups such as the *Chloroflexi*, *Planctomycetes*, WS1, *Bacteroidetes*, *Firmicutes*, JS1, OP11 and other taxa/divisions have been observed in methane seep sediments (1, 9, 11, 15, 17, 20, 22, 29, 31).

In this study, we found atypical microbial communities in a sediment core from a microbial mat site on a deep-sea methane seep in the Nankai Trough by quantitative PCR techniques and geochemical analyses, and then applied SSU rRNA gene tag-sequencing analysis. Pyrosequencing technique applied to SSU rRNA gene tag-sequencing analysis of marine and soil biospheres, including hydrocarbon seep sediments, retrieved more than 5,000 sequences and revealed a ‘rare biosphere’ (*e.g.*, 12, 18, 21, 25, 32, 35). Here, we applied this technique to elucidate the richness of the microbial diversity and rare members in atypical microbial communities of methane seep sediments. In this study, oligonucleotide primer sequences of 530F and 907R were also reexamined, and novel primer sets of 530F and 907R were applied to the amplification of SSU rRNA gene fragments from DNA assemblages. The tag-sequences obtained were used for statistic analyses that demonstrate the richness of the microbial diversity in methane seep sediments. Furthermore, we compared the diversity and richness of each phylogroup according to the depth of the methane seep sediment.

## Materials and Methods

### Site description, sampling and geochemistry of core sample

**Fig. 1:**
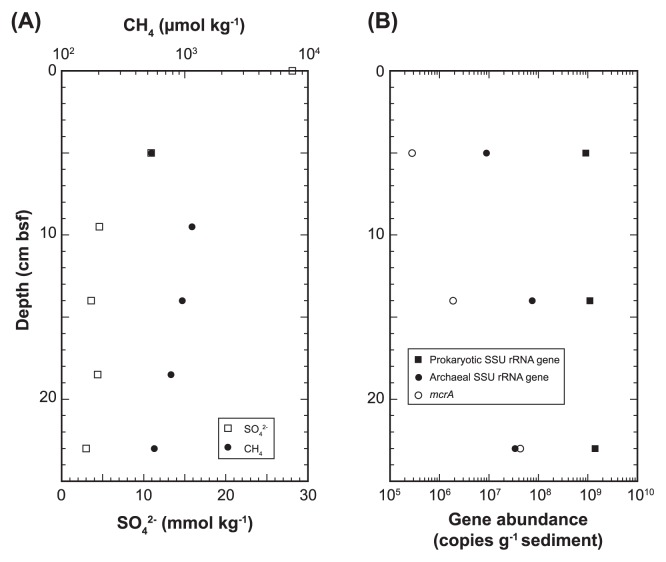
Concentrations of sulfate and methane (A), and abundances of genes for all prokaryotic and archaeal SSU rRNA, and *mcrA* (B) in sediment core 949C3. Methane concentration at sediment-water interface was not determined.

Sediment core 949C3 (25 cm in length) was taken by a push corer from a bacterial mat in an active methane seep site at the Omine Ridge (2,519 m below the sea surface) (33°7.33′N, 136°28.77′E), Nankai Trough off Kumano area with a manned submersible ‘*Shinkai 6500*’ (dive #6K949) on the YK06-03 cruise (April 2006) of *R/V Yokosuka* (JAMSTEC, Japan). The sediments consisted of blackish-gray sandy-silt and contained hydrogen sulfide. The core was subsampled by sterilized top-cut syringes and spatulas at 5 cm intervals on board, stored at −80°C for microbiology and used for interstitial water geochemistry. Sulfate concentration in core 949C3 decreased with increasing depth and more than 5.4×10^2^ μmol kg^−1^ methane was detected through the sediment core column (Fig. 1A). Geochemical data were provided by Toki *et al.* (personal communication) and will be published elsewhere.

### Nucleic acid extraction and quantitative PCR analyses

DNA was extracted from approx. 10 *g* sediments using an Ultra Clean Power Max Soil DNA Purification Kit (Mo Bio Laboratories, Carlsbad, CA, USA). The archaeal and total prokaryotic SSU rRNA genes from environmental microbial DNA were quantified using a quantitative fluorescent PCR method with a 7500 Real Time PCR System (PE Applied Biosystems, Foster City, CA, USA) described previously (38) with minor modification following Nunoura *et al.* (26). Quantitative PCR for *mcrA* with a primer set of ME3MF and ME2r’ was conducted using SYBR Premix Ex Taq II (Takara, Otsu, Japan) in the presence of MgCl_2_ (final 2.5 mM) as described previously (26).

### Reconstruction of primer set

In order to retrieve environmental SSU rRNA gene fragments from all phylogenetic groups (phylogroups), we surveyed 530F and 907R primer sites (19) corresponds to V4 and V5 regions in 105,346 bacterial and 6,517 archaeal SSU rRNA gene sequences by ARB version 20030822 in 2007 (23) (Table 1). Based on this survey, we reconstructed 7 and 6 primer sequences for 530F and 907R sites targeting all prokaryotic SSU rRNA genes (Table 2) and 3 and 5 primer sequences for 530F and 907R sites targeting the archaeal SSU rRNA gene (Table S1). Mixtures of 530F and 907R sites were prepared (Table 2 and S1) in order to broaden the taxonomic range of SSU rRNA genes and minimize PCR amplification bias. The mixing ratio for all prokaryotic primer sets was determined as follows: 1) ratio between *Archaea* and *Bacteria* should be 1:1; 2) based on the population of sequences covered by each primer sequence, predominant primer sequences were determined; 3) based on the number of mismatch positions between the predominant primer sequence and minor sequences, the coverage of each primer sequence ratio between the predominant primer and minor primers was assessed. The coverage of the primer set was reconfirmed at the end of 2011 targeting sequences longer than 1.2 and 0.9 kb in length of bacterial and archaeal sequences, respectively, in SILVA SSU Ref NR database (http://www.arb-silva.de/download/arb-files/) (30). The SILVA SSU Ref NR database is based on the full Ref 108 and HSM/MWM datasets with a 99% criterion applied to remove the redundant sequence described in http://www.arb-silva.de/projects/ssu-ref-nr/ (30). Group specific mismatches between 530F and 907R primer sequences, and the coverage of primer sequences targeting SSU rRNA gene sequences in SILVA SSURef database are summarized in Table 3, S2 and S3. The primer set for all prokaryotic SSU rRNA genes covers 92.6, 84.1 and 86.3% of bacterial, archaeal and eukaryotic SSU rRNA genes without mismatch residues, respectively.

### Amplification of SSU rRNA gene and pyrosequencing

DNA assemblages for SSU rRNA gene tag-sequencing analysis were extracted from sediments at 5 and 25 cm below the seafloor (cmbsf). PCR amplification of SSU rRNA gene fragments using the primer set of ‘530F’ and ‘907R’ described above was conducted under the following conditions. The PCR mixture contained 0.5 volumes of 2×GC buffer I for LA Taq polymerase (Takara), 0.1 U μL^−1^ LA Taq, 0.4 pmol μL^−1^ of each primer mixture and 1.6 nmol μL^−1^ dNTP. The DNA amplification condition was 5 min of denaturation at 96°C, and 25 cycles at 96°C for 20 s, 48°C for 70 s and 72°C for 30 s. Each of the ‘530F’ and ‘907R’ oligonucleotide primers harbored 24 bases of non-palindromic sequencing “key” sequences ‘CCATCTGTTCCCTCCCTGTCTCAG’ and ‘CCTATCCCCTGTTGCGTGTCTCAG’ for pyrosequencing at their 5′ terminal end, respectively. These primers were mixed as shown in Table 2 and applied to PCR amplification of SSU rRNA gene fragments. The amplified SSU rRNA gene fragments that were purified by agarose gel electrophoresis were applied to another 3 rounds of amplification to add the sequence for emulsion PCR and labeling biotin required for 454 pyrosequencing. The primers used in the other PCR amplification were adaptor A ‘CCATCTCATCCCTGCGTGTCCCATCTGTTCCCTCCCTGTCTCAG’ and adaptor B ‘BioTEG/CCTATCCCCTGTGTGCCTTGCCTATCCCCTGTTGCGTGTCTCAG’ to extend the “key” sequences described above. These adaptors are necessary to capture single-strand DNA for emulsion PCR. Primer sequence for pyrosequencing was ‘CCATCTGTTCCCTCCCTGTC’ encoded in adaptor A. The PCR mixture consisted of 0.1 volumes of 10×Ex Taq buffer, 0.05 U μL^−1^ of Ex Taq (Takara), 0.4 pmol μL^−1^ of each primer, 0.8 nmol μL^−1^ dNTP and 0.01 pmol SSU rRNA gene fragments. The amplification condition was 4 min of denaturation at 96°C, and 4 cycles at 96°C for 30 s, 64°C for 30 s and 72°C for 1 min. The biotin-labeled SSU rRNA gene fragments were purified by gel electrophoresis. The SSU rRNA gene fragments were analyzed by 454 pyrosequencer GS20 (Roche, Basel, Switzerland) in Takara. A gasket separated into 16 regions was used for pyrosequencing and we applied one sample per region in this study.

### Phylogenetic assignment and clustering of SSU rRNA gene fragments, and statistics

Sequences shorter than 90 bp were removed from the analysis. Sequencing tags were aligned using the partial order algorithm (POA) (SINA, http://www.arb-silva.de/aligner/) with a reference multiple alignment SILVA SSU Ref NR. Then, tags were clustered into operational taxonomic units (OTU) by 98% sequence identity using MOTHUR 3.6 with default parameters (9). The taxonomic position of each OTU was automatically assigned based on Blast analysis in the QIIME software package (6) using SILVA Ref NR as a reference dataset of SSU rRNA gene sequences. Sequences presenting with a relatively high E-value (>1.0E-30) or low identity (<90%) to best match the reference sequence were designated as other archaea or bacteria, and sequences that did not harbor significant identity to any reference sequences were excluded from the analysis. Alpha diversity indexes (rarefaction curves, Chao1, ACE, Shannon, Shannon evenness and Shimpson) in each of the libraries and taxa/divisions were also calculated using MOTHUR 3.6 (33).

### Accession numbers

Sequences and quality from pyrosequencing runs were deposited in the DDBJ/EMBL/GenBank database under accession number DRA000449.

## Results

### Quantitative PCR of genes for SSU rRNA and *mcrA*

The copy numbers of total prokaryotic and archaeal SSU rRNA genes in DNA assemblages from anoxic methane seep sediments ranged from 7.8×10^7^ to 1.2×10^8^ copies g^−1^ sediments and 1.4×10^6^ to 3.8×10^6^ copies g^−1^ sediment, respectively (Fig. 1B). The abundance of *mcrA*, a key gene of AOM, was 2.8×10^5^ to 4.3×10^7^ copy numbers g^−1^ sediment (Fig. 1B). Archaeal abundance was 1.0, 6.8 and 2.4% at 5, 15 and 25 cmbsf, respectively, in the SSU rRNA gene population, and the ratio of *mcrA* to archaeal SSU rRNA gene was 3.1, 2.5 and 126% at 5, 15 and 25 cmbsf, respectively.

### Microbial community composition in methane seep sediments

**Fig. 2:**
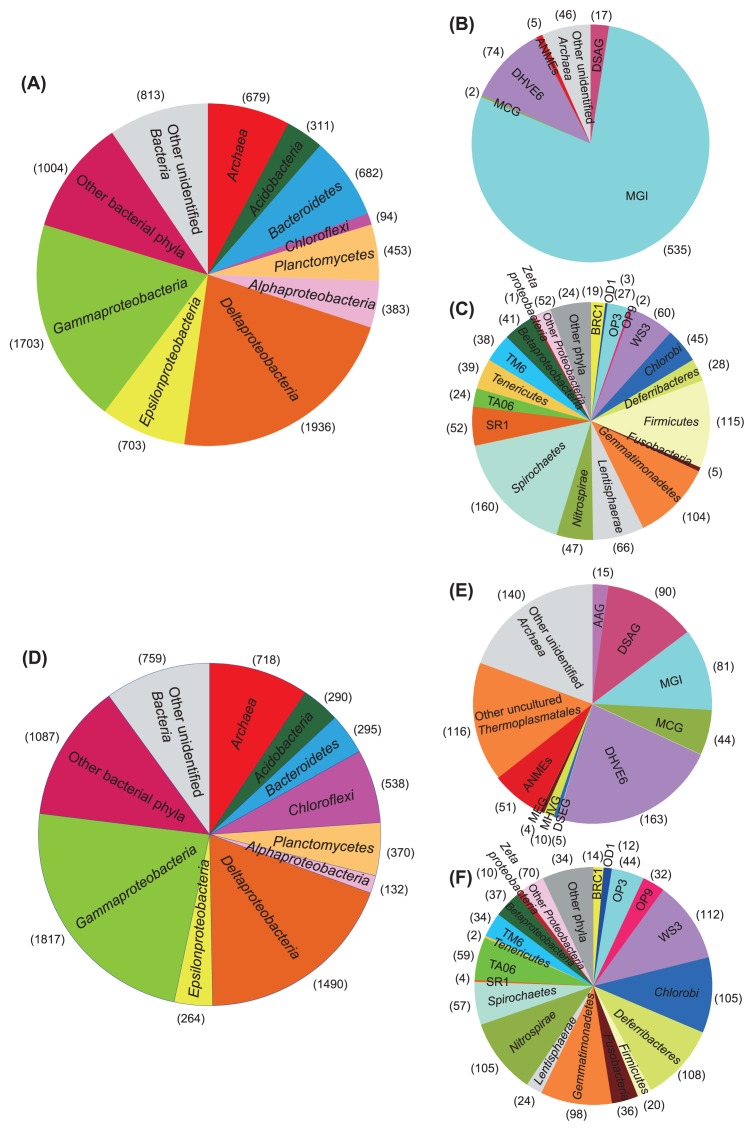
Composition of abundant phyla/classes/divisions in SSU rRNA gene communities at 5 and 25 cmbsf in methane seep sediment core 949C3 (A and D, respectively). Compositions of archaeal SSU rRNA gene tags at 5 and 25 cmbsf are presented in B and E, respectively, and minor bacterial populations presented as ‘other bacterial phyla’ in A and D are demonstrated in C and F, respectively. ‘Other phyla’ in C and F include *Armatimonadetes* (OP10), OP11, TM7, *Chlamidiae*, *Elusimicrobia* (TM1), *Fibrobacteres* and *Verrucomicrobia* while OP11 was detected only at 5 cmbsf. Numbers in parentheses indicate tag numbers for each phylum/class/division.

Each 8,709 and 7,690 SSU rRNA gene tag-sequence was obtained from methane seep sediments at 5 and 25 cmbsf, respectively. These 679 and 718 tag-sequences at 5 and 25 cmbsf, respectively, were identified as the archaeal SSU rRNA gene (Fig. 2).

**Fig. 3:**
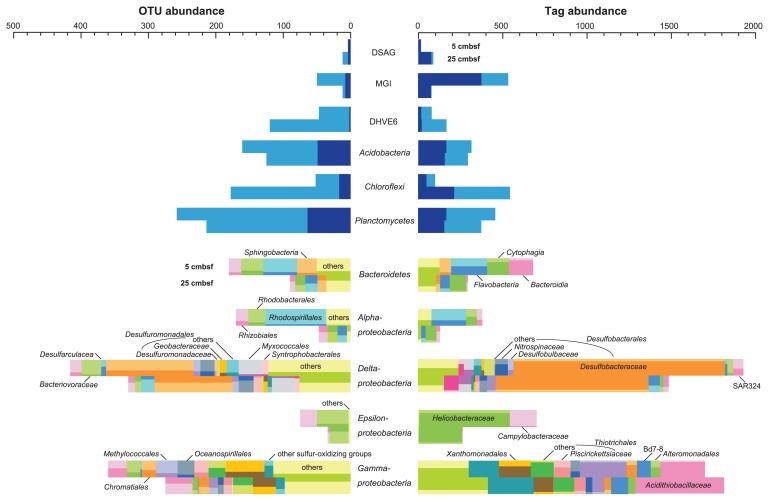
Abundance of shared tags and OTUs in each taxon/division between depths of 5 and 25 cmbsf in sediment core 949C3, and the order or family-level composition of bacterial major phyla/classes based on top hit sequences of each tag sequence in Blast analysis. Darker area for each taxon/division indicates shared population.

The phylum *Proteobacteria* shared 55.3 and 49.7% of total tag-sequences from sediments at 5 and 25 cmbsf, respectively (Fig. 2). *Deltaproteobacteria* and *Gammaproteobacteria* were the predominant proteobacterial classes at both depths. Sequences related to the sulfate-reducing family *Desulfobacteraceae* predominated in the deltaproteobacterial populations (Fig. 3 and Table S4). Among the gamma-proteobacterial sequences, sequences related to sulfur oxidizers were more abundant than those related to heterotrophs. The dominant gammaproteobacterial sulfur-oxidizing family detected in this study was *Acidithiobacillaceae*, and sequences similar to other sulfur-oxidizing families, such as *Ectothiorhodospiraceae* and *Chromatiaceae* in the *Chromatiales*, *Piscirickettsiaceae* and *Thiotrichaceae* in the *Thiotrichales*, and the *Thiohalophilus*-related group were also detected. Sequences related to the methanotrophic gammaproteobacteial order *Methylococcales* were also found as one of the dominant members of the gammaproteobacterial sequences at 5 cmbsf and its abundance remarkably decreased at 25 cmbsf (Fig. 3 and Table S4). Abundance of epsilon-and alphaproteobacterial sequences decreased with the depth increased. The dominant populations in *Epsilonproteobacteria* were closely related to sulfur-oxidizing genera such as *Arcobacter* in *Campylobacteraceae*, and *Sulfurimonas* and *Sulfurovum* in *Helicobacteraceae*. The predominance of *Campylobacteraceae* sequences in the epsilonproteobacterial tags was only found at 5 cmbsf (Fig. 3 and Table S4). As minor populations among proteobacterial sequences, we found 1 and 10 sequences related to the marine Fe-oxidizing ‘*Zetaproteobacteria*’ from 5 and 25 cmbsf, respectively (7) (Fig. 2). SSU rRNA gene similarity between “*Mariprofundus ferrooxydans*” and four zetaproteobacterial OTUs obtained in this study (P9_5856, P9_1214, P9_1934 and P9_3677) ranged from 92 to 97%. Most of the zetaproteobacterial SSU rRNA gene sequences have been detected from hydrothermal environments with a few exceptions, such as the brine-seawater interface in the Red Sea (8), a salt marsh environment (24) and biocorroding microbiota in coastal seawaters (7). This is the first observation of zetaproteobacterial sequences in methane seep sediments. The other abundant bacterial phyla were *Acidobacteria*, *Bacteroidetes*, *Chloroflexi* and *Planctomycetes*, up to 7.0% of bacterial sequences in each depth. In addition to these phyla, *Armatimonadetes* (OP10), *Actinobacteria*, *Chlamidiae*, *Chlorobi*, *Deferribacteres*, *Elusimicrobia* (TM1), *Firmicutes*, *Fusobacteria*, *Gemmatimonadetes*, *Lentisphaerae*, *Nitrospirae*, *Spirochaetes*, *Tenericutes* and *Verrucomicrobia* were detected as minor populations (Fig. 2 and Table S4). Tag-sequences related to previously uncultivated candidatus bacterial divisions such as BRC1, OD1, OP3, OP9, OP11, SR1, TA06, TM6, TM7 and WS3 were also observed in minor populations.

In contrast to bacterial populations, significant differences in archaeal communities were observed between the depths of 5 and 25 cmbsf (Fig. 2 and 3). In this study, groupings of uncultivated archaeal lineages followed the SILVA database. The Deep Sea Euryarchaeotic Group (DSEG) in the SILVA database includes Deep-sea Hydrothermal Vent Euryarchaeotic Group 8 (DHVEG 8) and a part of DHVEG 4, the DHVEG 6 (SILVA) contains DHVEG 5, and the Miscellaneous Euryarchaeotic Group (MEG) (SILVA) also harbors DHVEG 3 and a part of DHVEG 4. In the sediments at 5 cmbsf, the MGI predominated with 78.8% of archaeal sequences, and the next abundant group was the DHVEG 6 with 10.9% of archaeal sequences. On the other hand, at a depth of 25 cmbsf, the abundance of MGI-related sequences decreased to 11.3%, and those of other uncultivated lineages such as the DHVEG 6, DSAG and other uncultivated *Thermoplasmatales* increased to more than 12.5% of archaeal sequences. The unexpected dearth of potential anaerobic methanotrophs (ANME) was observed even at 25 cmbsf. At 5 cmbsf, only one each of the clones of ANME-1 and Lost City *Methanosarcinales* sequences and 3 ANME-3/*Methanococcoides* sequences was found, and 2 ANME-1, 13 ANME-2a, 14 ANME-2c and 21 ANME-3/*Methanococcoides* sequences were detected at 25 cmbsf. The ANME-3 and *Methanococcoides* methanogens could not be distinguished by short tag-sequences. In addition, the DSEG, MEG, MCG, Ancient Archaeal Group (AAG), Marine Hydrothermal Vent Group (MHVG) and Marine Benthic Group D (MBGD) were detected as minor populations in the archaeal tag population (Table S4).

### Diversity and richness

**Fig. 4:**
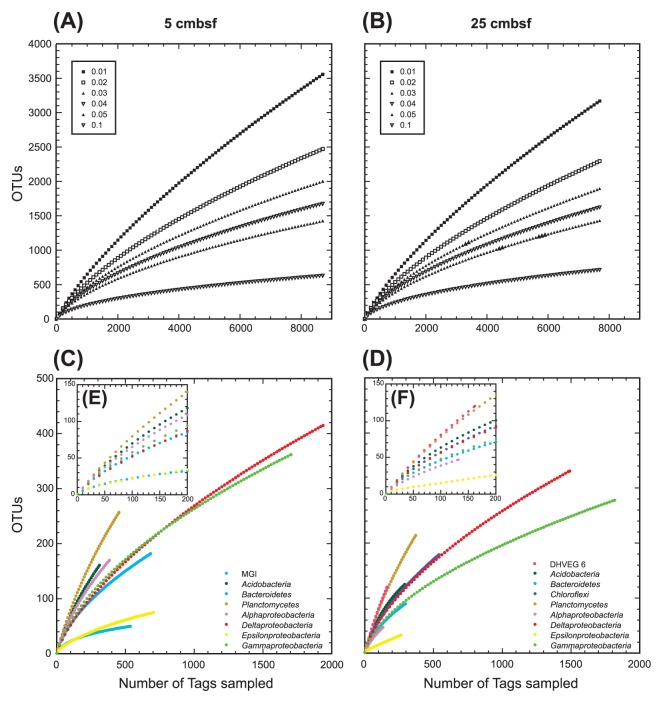
Rarefaction analysis for methane seep sediment samples from 5 and 25 cmbsf of sediment core 949C3 based on MOTHUR program. Rarefaction of whole microbial community is shown for OTUs with differences that do not exceed 1, 2, 3, 4, 5 and 10% (A and B). C and D show taxon/division-specific rarefaction for OTUs with <2% dissimilarity. E and F show rarefaction at <200 tags in C and D, respectively.

The richness of microbial diversity in each sample was assessed by rarefaction curves, and Chao1 and ACE indexes were calculated by MOTHUR. Both rarefaction curves of 5 and 25 cmbsf present with similar diversity richness and revealed that we could not retrieve whole microbial diversity by this tag-sequencing analysis (Fig. 4). We identified al least 2,467 and 2,290 OTUs (<2% dissimilarity) from sediments at 5 and 25 cmbsf, respectively. Chao1 index at 5 and 25 cmbsf was 6,558 and 6,373, and ACE index at 5 and 25 cmbsf was 11,118 and 11,269, respectively (Table S5).

In this study, we also examined the microbial diversity of each abundant archaeal and bacterial taxon/division that harbors more than 100 tags, such as the MGI, DHVEG 6, *Acidobacteria*, *Bacteroidetes*, *Chloroflexi*, *Planctomycetes*, and *Alpha-*, *Gamma-*, *Delta-* and *Epsilonproteobacteria* (Fig. 4 and Table S5). The Shannon index of diversity varied from 1.68 to 5.21 in bacterial taxa and 2.22 to 4.65 in archaeal divisions while similar uniformity of the population was observed in both domains; 0.55 to 0.94 and 0.83 to 0.97 of Shannon evenness among bacterial and archaeal taxa/divisions, respectively (Table S5). The highest diversity was observed in the *Planctomycetes* population from both libraries, and the diversity of DHVEG 6 at 25 cmbsf was comparable to that of *Planctomycetes* (Fig. 4 and Table S5), while the archaeal diversity in previous studies was usually lower than bacterial diversity (1, 2, 11, 31). Both the diversity and richness of *Epsilonproteobacteria*, and MGI archaea were significantly smaller than in other taxa/divisions.

## Discussion

### Function and adaptation of the dominant microbial components in tag-sequencing

In order to know the distribution patterns within each dominant taxon/division along the depth, we examined the abundance of shared OTUs and tags in each taxon/division between sediments at 5 and 25 cmbsf (Fig. 3 and Table S4). Shared tags and OTUs at both depths suggest the presence of species or strains that may grow in both environments. In tag populations of *Desulfobacteraceae*, *Helicobacteraceae* and some gammaproteobacterial groups, most of the tags were shared at both depths while the abundance of shared OTUs in each OTU population was relatively low. This pattern indicates the presence of a depth-specific highly diverse minor population in these taxa. Low abundance of shared OTUs and shared tags in both depths was observed in the DHVEG 6 and *Planctomycetes* populations. The trend could be explained by the markedly high OTU richness (Fig. 4 and Table S5), and the presence of adapted populations in each environment. In tag populations of the DSAG, MGI and *Flavobacteria*, tag numbers at the two depths differed significantly, and most of the tags in the smaller tag population were also found in the larger tag population at other depths. The results suggest that specific populations in each class/division can survive and grow in their hostile environments in a relatively small population, such as the oxidative stress-tolerant population of anaerobic DSAG in a relatively oxidative environment at 5 cmbsf, and reductive stress-tolerant population of obligately aerobic or facultatively anaerobic MGI archaea at 25 cmbsf. In the epsilon-proteobacterial population, *Campylobacteraceae* tags almost disappeared at 25 cmbsf while *Helicobacteraceae* tags still dominated at 25 cmbsf. Considering the versatile energy metabolisms observed in both families (5), such family-specific distribution found in this study is unexpected. Among bacterial phyla that harbor fermentation metabolism such as *Acidobacteria*, *Bacteroidetes* and *Chloroflexi*, each phylum presents with different distribution patterns. The abundance of *Acidobacteria* was constant along the depth while that of *Bacteroidetes*, especially the classes *Bacteroidia* and *Flavobacteria*, decreased and *Chloroflexi* increased at 25 cmbsf. The growth of *Bacteroidetes* might be inhibited by higher sulfide concentration at 25 cmbsf, and *Chlorofelxi* species may prefer more reduced environments than *Acidobacteria* and *Bacteroidetes* species.

### Abundance and functions of ANME and other *Archaea*

The proportion of archaeal SSU rRNA gene tag sequences was 7.8 and 9.3% in the tag populations from 5 and 25 cmbsf, respectively, while that estimated by quantitative PCR for SSU rRNA genes was 1.8% and 2.4%, respectively. The archaeal population in all prokaryotic SSU rRNA genes shown by tag-sequencing is different from that observed by quantitative PCR, but the trend for the abundance of *Archaea* in the sediments at 25 cmbsf to be larger than that at 5 cmbsf is consistent in both analyses. A similar trend between quantitative PCR and SSU rRNA gene clone analysis using the same primer sets was also observed in other experiences (13).

Comparing the tag-populations from the two depths, the abundance of tags from MGI decreased and those from DHVEG 6 and DSAG increased at 25 cmbsf. At 5 cmbsf, DHVEG 6 shared relatively high abundance while DSAG was just a minor population (Fig. 2 and Table S4). DHVEG 6 has been detected from relatively anoxic terrestrial soils and marine sediments (10, 37, 39), and DSAG has been observed in anoxic marine sediments from diverse environments, such as the ambient seafloor, methane seep and hydrothermal field (37, 39). On the other hand, inhabitance of DHVEG 6 in shallower sediments than DSAG was also found in the archaeal community structure associated with ambient sediments in a hydrothermal field (27). These distribution patterns suggest that DHVEG 6 can grow in relatively oxidative environments while other anaerobic archaeal groups such as DSAG prefer more reductive environments.

The geochemical profile of sulfate and methane concentrations in sediment core 949C3 suggested the occurrence of AOM (Fig. 1A), and SSU rRNA gene tag sequencing revealed the presence of ANME (Fig. 2). Copy numbers of *mcrA* at 25 cmbsf were about 150-fold larger than those at 5 cmbsf, and the ratio of *mcrA* to the prokaryotic SSU rRNA gene copy number was 0.06 and 3.1% at 5 and 25 cmsf, respectively (Fig. 1B). On the other hand, ANME-related sequences shared 0.07 and 0.66% of SSU rRNA gene tags at 5 and 25 cmbsf, respectively, and no SSU rRNA gene tags from methanogens distant from ANME were detected in the tag libraries. These results suggest that the abundance of the ANME SSU rRNA gene sequences was likely underestimated in the SSU rRNA gene tag population although no mismatch residue was found between ANME SSU rRNA gene sequences in the public database and primer sequences used in this study (Table 1 and 2).

### Overview of microbial community in methane seep sediments

Dominant archaeal phylogroups found in tag populations were not related to the sequences from ANME or methanogens, and could not be signatures of methane seep environment as described above; however, the high abundance of *mcrA* in the archaeal population and geochemical profiles suggest the occurrence of AOM in sediment core 949C3 (Fig. 1, 2 and Table S4). Furthermore, the high tag abundance of sulfur oxidizers belong to *Gammaproteobacteria* and *Epsilonproteobacteria*, and that of sulfate-reducing *Deltaproteobacteria* in SSU rRNA gene tag populations at both 5 and 25 cmbsf suggests the co-occurrence of sulfur oxidation and sulfate reduction through the core column (Fig. 3). Consequently, we conclude that the microbial communities found in this study were associated with methane seep sediments but not ambient seafloor sediments.

In the previous study of hydrocarbon seep deep-sea sediment in the Gulf of Mexico by SSU rRNA gene V6 region tag-sequencing analysis, community structures of sediments at 13.5 cmbsf from within and outside a mat formation site were examined (21). In the case of the Gulf of Mexico hydrocarbon seep site, higher AOM activity and low microbial diversity was observed in sediments within a mat formation while higher diversity was observed in sediments from outside a mat formation at the same seep site. Higher microbial diversity in sediments from outside the mat formation was also confirmed by rRNA cDNA clone analysis. Compared with the microbial diversity at the Gulf of Mexico site, the microbial diversity found in this study (Chao1 6,558 and 6,374) is similar to that from outside the mat formation (Chao1 <5,909) rather than that from the mat formation (Chao1 <58) at the Gulf of Mexico site, although the geochemical profile of core 949C3 is similar to that in the core from the microbial mat formation. The richness of the anoxic methane seep sediment observed in the rarefaction curves and the richness of anoxic methane seep sediment in this study is likely similar to those in soils (32) and deep-sea hydrothermal environments (12, 35).

Unexpectedly, the abundance of genes for archaeal SSU rRNA and *mcrA* in the methane seep sediments (core 949C3) was apparently smaller than those in previously analyzed methane seep sediments (core 744C1) taken from the other microbial mat formation site on the same ridge in April 2003; archaeal SSU rRNA gene abundance at 5 cmbsf was 9.0×10^6^ and 4.6×10^8^ copies g^−1^ sediment and at 25 cmbsf was 2.9×10^9^ and 3.4×10^7^ copies g^−1^ sediment, in cores 949C3 and 744C1, respectively, and *mcrA* gene abundance at 5 cmbsf was 2.8×10^5^ and 4.6×10^7^ copies g^−1^ sediment, and at 25 cmbsf was 3.4×10^7^ and 2.6×10^9^ copies g^−1^ sediment, in cores 949C3 and 744C1, respectively (26, 28). In addition, quantitative PCR analysis revealed that the archaeal SSU rRNA gene population in all prokaryotic SSU rRNA genes in core 949C3 (1.7 to 6.7%) was smaller than that in core 744C1 (27 to 39%). Furthermore, the predominance of MGI in the archaeal population at 5 cmbsf in core 949C3 was observed in SSU rRNA gene tag-sequencing while MGI was not detected in archaeal SSU rRNA gene clone analysis at the same depth in core 744C1 (28). On the other hand, the geochemical profile of core 744C1 also suggests the occurrence of AOM, as in the case of core 949C3, while core 744C1 bore lower concentrations of methane; methane concentrations in core 949C3 (5.4×10^2^-1.2×10^3^ μmol kg^−1^) were higher than those of 744C1 (4.2–6.0×10^1^ μmol kg^−1^) (26, 36). The typical geochemical profiles of active AOM and increasing *mcrA* population with increasing depth in core 949C3 (Fig. 1) suggest the presence of a typical mature AOM microbial community predominated by ANME and *Deltaproteobacteria* (16) below the sediments analyzed in this study. Accordingly, we conclude that ANME communities in core 949C3 have not matured yet. We assume two potential geological reasons for the undeveloped ANME community in core 949C3, although we cannot conclude the geological systems of the methane seep site without long-term monitoring of methane flux and/or other hydrological survey at the methane seep site. One potential reason is the recent upwelling of a high concentration of methane in the sediments, and the AOM-dependent microbial community was at the developmental stage. In this case, a mature AOM microbial community would occur just beneath the seafloor, as in the case of core 744C1. The other is that the input of oxidative seawater inhibits the development of AOM suggested by the co-occurrence of potential sulfur oxidizers and sulfate reducers at 25 cmbsf.

From another aspect, the microbial communities analyzed are likely analogues of microbial communities just beneath the seafloor under the microbial mat formation site that harbors typical mature AOM-dependent microbial communities. In typical methane seep sediments presenting geochemical signatures of sulfate reduction and methane oxidation, the transition of the sulfur-oxidizing community to sulfate-reducing ANME community occurs within a few cm, and the ANME community predominates in the core column (4, 11, 15, 17, 29). Thus, it is intractable to observe such community transition because of the difficulty of sampling such thin layers from the sediment core. In contrast to such typical methane seep sediments, the microbial communities observed in this study were not predominated by the ANME community, although typical geochemical signatures of sulfate reduction and methane oxidation were observed. By demonstrating SSU rRNA gene tag-sequencing analysis of atypical microbial communities associated with methane seep sediments, we can observe the unseen distribution pattern of microbes in relatively oxidative environments, especially the family-specific distribution patterns found in the *Epsilonproteobacteria* (Fig. 3).

## Supplementary Material



## Figures and Tables

**Table 1:** Comparison of sequences between previouly constructed primers and sequences in database at 2007

	Seaquence (5′ to 3′)	No. of sequences		Coverage

		*Archaea*	*Bacteria*	
530F	GTGCCAGCMGCCGCGG			
1	GTGCCAGCAGCCGCGG	113	101,157	Most of the *Bacteria*, *Methanococcales*, some uncultured *Archaea*
2	GTGCCAGCCGCCGCGG	3,518	32	diverse uncultured Archaea*
3	GTG**T**CAGCCGCCGCGG	2,087	0	*Crenarchaeota*
4	GTG**G**CAGCCGCCGCGG	291	0	*Archaeoglobales*, *Thermococcales*, some *Korarchaeota*
5	**C**TGCCAGCCGCCGCGG	20	0	*Methanopyrus*, uncultured *Euryarchaeota*
6	**T**TGCCAGCCGCCGCGG	49	0	some uncultured *Halobacteriales* & diverse uncultured *Euryarchaeota*
7	GTGCCAGCAGC**T**GCGG	0	390	*Chlamydiaceae* & OP5 & diverce bacterial groups
8	GTGCCAG**A**AGC**GT**CGG	0	59	OP11
9	GTGCCAGCAG**T**CGCGG	0	244	OP11 & OD1 & diverce bacterial groups
10	GTGCCAG**A**AGCC**T**CGG	0	32	OP11 & WS6 & OD1
11	GTGCCAG**A**AG**AAT**CGG	0	12	WS6
12	GTGCCAG**A**AGC**AT**CGG	0	33	WS6 & OD1
13	GTGCCAGCAGC**A**GCGG	0	132	WS6 & OP11 & OD1
14	GTGCCAGCAG**GA**GCGG	0	15	OD1
15	GTG**G**CAG**TC**GCC**A**CGG	2	0	*Nanoarchaeota*
		6,080	102,106	
907R	CCGTCAATTCMTTTRAGTTT			
1	CCGTCAATTCCTTTGAGTTT	0	80,234	Most of *Bacteria*
2	CCGTCAATTCCTTTAAGTTT	37	889	some *Korarchaeota* & diverse archaeal group
3	CCGTCAATTCATTTGAGTTT	1	14,881	diverse bacterial groups
4	CCG**C**CAATTCCTTTAAGTTT	5,877	8	most of Archaea*
5	CCG**C**CAATT**T**CTTTAAGTTT	73	0	SAGMEG & diverse archaeal group
6	CCG**C**CAATTCCTTTGAGTTT	9	740	some *Desulfurococcales*species, *Aquificae*, *Thermomicrobia* & OP11
7	CCGTCAATTCC**C**TTGAGTTT	0	107	OP1 & diverse bacterial groups
8	CCGTCAATTCCTTTA**T**GTTT	0	169	TM7 & diverse bacterial groups
9	CCG**C**CAATTCCTT**A**AAGTTT	40	0	uncultured *Halobacteriales* & several uncultured *Archaea*
10	CCG**C**CAATTCC**C**TTAAGTTT	43	0	DSEG & several uncultured archaeal group
11	CCGTC**T**ATTCCTTTGAGTTT	0	1,809	*Desulfurobacteriaceae* & OD1
12	CCGTCAATTCATTTAAGTTT	3	24	Ancient Archaeal Group, uncultured *Gammaproteobacteria*
13	CCGTCAATTCCTTCAAGTTT	31	2	almost *Korarchaeota*
14	CCG**C**C**T**ATTCCTTTAAGTTT	16	0	*Nanoarchaeota*
15	CCG**C**CAATTCCTTCAAGTTT	7	0	diverse uncultured archaeal group
16	CCG**C**CAATTCCTTTGA**A**TTT	0	11	OP11
17	CCGTCAATTCCTTTGAG**C**TT	0	83	diverse bacterial groups
18	CCG**C**CAATTCCTTTAAGT**A**T	6	0	diverse archaeal groups
19	CCG**C**CTATTCCTTTGAGTTT	0	21	OD1 & some bacterial groups
20	CCG**C**C**T**AT**C**CCTTTGAGTTT	0	11	OP11
21	CCG**C**CAATTC**G**TTTGAGTTT	0	26	OP11
22	CCG**C**CAATTCATTTGAGTTT	0	27	WS6
23	CCGTCAATT**T**CTTTGAGTTT	0	2,580	Some of *Deltaproteobacteria* & diverse bacterial groups
24	CCGTCAATTC**T**TTTGAGTTT	0	572	*Planctomycetes, Chlomadiae*
25	CCGTCAATTC**G**TTTGAGTTT	0	142	EM19, uncultured *Chloroflexi*
26	CCG**C**CAATTCCCTTAAGTTT	43	0	SAGMEG
		6,186	102,336	

* All of the ANME related seqences are included in these group without any mismatch residues.

**Table 2:** Prokaryotic SSU rRNA gene primer sequences constructed in this study

Name	Sequence (5′-3′)	Group coverage	Proportion in mixtures
Prokaryotic set			
30F mixture			
Bac 530F	GTGCCAGCAGCCGCGG	1	30
Arch 530F	GTGBCAGCCGCCGCGG	2, 3, 4	24
Arch2 530F	YTGCCAGCCGCCGCGG	5, 6	6
Bac2 530F	GTGCCAGCAGCWGCGG	7, 13, 14	1
Bac3 530F	GTGCCAGCAGTCGCGG	9	1
Bac4 530F	GTGCCAGAAGMMTCGG	10, 11, 12, (8)	1
Nano 530F	GTGGCAGTCGCCACGG	15	3
907R mixture			
Uni 907R	CCGYCAATTCMTTTRAGTTT	1, 2, 3, 4, 6, 12, 19, 22, (8, 17, 18, 23, 24, 25)	20
DeepAB 907R	CCGYCTATTCCTTTGAGTTT	11, (14), 20	1
SAG-Del 907R	CCGYCAATTTCTTTRAGTTT	5, 23	1
DeepAB2 907R	CCGYCAATTCCCTTRAGTTT	7, 10, 26	1
Arch2 907R	CCGYCAATTCCTTMAAGTTT	9, 13, 15	1
OP11 907R	CCGCCAATTCCTTTGAATTT	16, 21	1

Numbers in group coverage are refered from Table 1.

Groups in parentheses harbor 1 base mismatch with primer sequence.

**Table 3:** Coverage of 530F and 907R primers targeting SILVA SSURef database at the end of 2011. In the database, we identified archaeal 13,022, badterial 282,285 and eukariotic 36,054 sequences that harbor both 530F and 907R regions

(A) Number of sequences without mismatch residues compared to 530F primers.			

530F primers	*Archaea*	*Bacteria*	*Eukarya*
Arch_530F	12,062	63	78
Arch2_530F	90	3	0
Nano_530F	38	0	0
Bac_530F	214	271,887	33,165
Bac2_530F	0	1,031	502
Bac3_530F	0	562	38
Bac4_530F	0	188	1

Total	12,404	273,734	33,784

Coverage (%) without mismatch residues	95.3	97.0	93.7

(B) Number of sequences with 1 mismatch residues compared to 530F primers.			

530F primers	*Archaea*	*Bacteria*	*Eukarya*

Arch_530F	298	490	49
Arch2_530F	12	222	16
Nano_530F	9	0	0
Bac_530F	19	3,979	1,056
Bac2_530F	7	174	39
Bac3_530F	2	151	39
Bac4_530F	0	169	14

Total	347	5,185	1,213

Coverage (%) with 1 mismatch residue	2.7	1.8	3.4

(C) Number of sequences without mismatch residues compared to 907R primers.			

907R primers	*Archaea*	*Bacteria*	*Eukarya*

Uni_907R	11,166	256,453	27,714
DeepAB_907R	0	3,835	0
SAG-Del_907R	158	6,020	5,679
DeepAB2_907R	138	428	25
Arch2_907R	159	4	368
OP11_907R	0	7	0

Total sequences	11,621	266,747	33,786

Coverage (%) without mismatch residues	89.2	94.5	93.7

(D) Number of sequences with 1 mismatch residues compared to 907R primers.			

907R primers	*Archaea*	*Bacteria*	*Eukarya*

Uni_907R	363	7,790	622
DeepAB_907R	1	760	31
SAG-Del_907R	17	437	420
DeepAB2_907R	27	225	7
Arch2_907R	13	256	72
OP11_907R	6	279	0

Total sequences	427	9,747	1,152

Coverage (%) with 1 mismatch residue	3.3	3.5	3.2
