# Supplementary material for: Microbial Diversity in Deep-sea Methane Seep Sediments Presented by SSU rRNA Gene Tag Sequencing
Source: Microbes Environ. 2012 Apr 18;27(4):382–90. doi: 10.1264/jsme2.ME12032 (PMC4103545; doi:10.1264/jsme2.ME12032)
Supplement: Supplementary file 1 [file 27_382_s1.pdf]

Table S1. Archaeal SSU rRNA gene primer sequences constructed in this study

Name	Sequence (5' - 3')	group coverage	Proportion in mixtures
Archaeal set			
530F mixture (Arch)			
Arch 530F	GTGBCAGCCGCCGCGG	2, 3, 4	24
Arch2 530F	YTGCCAGCCGCCGCGG	5, 6	6
Nano 530F	GTGGCAGTCGCCACGG	15	3
907R mixture (Arch)			
Arch 907R	CCGCCAATTCCTTTAAGTTT	4	10
SAGMEG 907R	CCGCCAATTTCTTTAAGTTT	5	1
Arch2 907R	CCGYCAATTCCTTMAAGTTT	9, 13, 15	1
DSEG SAGMEGB 907R	CCGCCAATTCCCTTAAGTTT	10, 30	1
Nano 907R	CCGCCTATTCCTTTAAGTTT	14	1

Archaeal set was not used in this study.

Table S2. Comparison of sequences between consensus sequences in Table 1 and SSU rRNA gene sequence in SILVA SSURef database in 2011. In the database, we identified archaeal 13022, badterial 282285 and eukariotic 36054 sequences that harbor both 530F and 907R regions.

(A)

	Number of sequences	Number of sequences without mismatch residues compared to 530F primers															Total sequences	Coverage (%)
	harbor 530F region	1	2	3	4	5	6	7	8	9	10	11	12	13	14	15		
Archaea	13022	214	6980	4724	358	35	55	0	0	0	0	0	0	0	0	38	12404	95.2
Bacteria	282285	271887	62	0	1	1	2	557	69	562	120	5	63	474	73	0	273876	97.0
Eukarya	36054	33165	78	0	0	0	0	496	0	38	1	0	0	6	0	0	33784	94.0

(B)

	Number of sequences	Number of sequences without mismatch residues compared to the 907R consensus seuqences																										Total sequences	Coverage (%)
	harbor 907R region	1	2	3	4	5	6	7	8	9	10	11	12	13	14	15	16	17	18	19	20	21	22	23	24	25	26		
Archaea	12404	1	82	1	11052	155	17	0	1	87	138	0	10	59	63	13	0	0	17	0	0	0	0	0	0	0	138	11834	90.1
Bacteria	273876	215442	1104	38076	11	0	1739	422	573	0	0	3732	21	2	0	0	7	266	0	103	46	39	63	5983	1339	238	0	269206	95.4
Eukarya	33784	13	27652	0	48	8	0	0	4	0	0	0	1	363	1	2	0	0	0	0	0	0	0	3	0	0	0	28095	77.9

Table S3. Coverage of 530F and 907R primers targeting SILVA SSURef database at the end of 2011. In the database, we identified archaeal 13022, bacterial 282285 and eukaryotic 36054 sequences that harbor both 530F and 907R regions.

(A) Number of sequences without mismatch residues compared to 530F primers used in archaeal set

	Archaea	*Bacteria*	*Eukarya*
Arch_530F	12062	63	78
Arch2_530F	90	3	0
Nano_530F	38	0	0
Total	12190	66	78
Coverage (%) without mismatch residues	93.6	0.0	0.2

(B) Number of sequences with 1 mismatch residues compared to 530F primers used in archaeal set

	Archaea	*Bacteria*	*Eukarya*
Arch_530F	298	490	49
Arch2_530F	12	222	16
Nano_530F	9	0	0
Total	319	712	65
Coverage (%) with 1 mismatch residue	2.4	0.3	0.2

(C) Number of sequences without mismatch residues compared to 907R primers used in archaeal set

	Archaea	*Bacteria*	*Eukarya*
Arch_907R	11052	11	48
Arch2_907R	159	4	368
SAGMEG_907R	155	0	8
DSEG_SAGMEGB_907R	138	0	0
Nano_907R	63	0	1
Total	11567	15	425
Coverage (%) without mismatch residues	88.8	0.0	1.2

(D) Number of sequences without mismatch residues compared to 907R primers used in archaeal set

	Archaea	*Bacteria*	*Eukarya*
Arch_907R	357	15	37
Arch2_907R	13	256	72
SAGMEG_907R	16	1	5
DSEG_SAGMEGB_907R	26	0	0
Nano_907R	27	17	31
Total	439	289	145
Coverage (%) with 1 mismatch residue	3.4	0.1	0.4

Table S4. Tag and OTU abundance of each taxon/division in SSU rRNA gene community structures at 5 and 25 cmbsf in methane seep sediment core 949C3, and abundance of shared tags and OTUs between the two depths. Characterization of each tag-sequence was based on top hit sequences of each tag sequence in Blast analysis. Tags with more than 98% identitiy were assinged as same OTU.

	Tags				OTUs			
	5 cmbsf		25 cmbsf		5 cmbsf		25 cmbsf	
	Total	Shared	Total	Shared	Total	Shared	Total	Shared
Archaea								
Ancient Archaeal Group (AAG)	0	0	15	0	0	0	6	0
Deep Sea Arcaheal Group (DSAG)	17	17	90	78	4	4	12	4
Marine Group I (MGI)	535	376	81	77	50	8	12	8
Miscellaneous Crenarchaeotic Group (MCG)	2	2	44	14	2	2	16	2
Deep Sea Hydrothermal Vent Euryarchaeotic Group 6 (DHVEG 6)	74	17	162	2	47	2	120	2
Deep Sea Euryarchaeotic Group (DSEG)	0	0	5	0	0	0	5	0
Marine Hydrothermal Vent Group (MHVG)	0	0	10	0	0	0	8	0
Miscellaneous Euryarchaeotic Group (MEG)	0	0	4	0	0	0	4	0
ANMEs	5	3	51	21	3	1	7	1
uncultured *Thermoplasmatales*	0	0	116	0	0	0	46	0
other archaea	46	14	140	22	26	2	81	2
Total (*Archaea*)	679	429	718	214	132	19	317	19
Bacteria								
Acidobacteria	311	167	290	158	161	49	125	49
Armatimonadetes (OP10)	1	0	6	0	1	0	6	0
Bacteroidetes								
Bacteroidia	143	124	9	4	18	3	8	3
Cytophagia	130	100	99	92	33	9	15	9
Flavobacteria	214	112	57	45	50	9	18	9
Sphingobacteria	68	11	23	12	29	4	13	4
other *Bacteroidetes*	127	59	107	71	50	12	36	12
Chlamydiae	2	0	3	0	2	0	3	0
Chlorobi	45	29	105	84	18	7	17	7
Chloroflexi	94	48	538	212	52	17	178	17
Deferribacteres	28	14	108	69	23	11	38	11
Elusimicrobia (TM1)	4	0	8	0	4	0	6	0
Fibrobacteres	6	1	9	1	4	1	7	1
Firmicutes	115	19	20	2	24	2	14	2
Fusobacteria	5	4	36	25	5	4	8	4
Gemmatimonadetes	104	48	98	55	46	12	36	12
Lentisphaerae	66	13	24	12	28	5	17	5
Nitrospirae	47	20	105	45	31	12	55	12
Planctomycetes	453	168	370	156	258	64	214	64
Proteobacteria								
Alphaproteobacteria								
Rhizobiales	34	12	22	8	18	5	13	5
Rhodobacterales	62	33	64	55	26	5	14	5
Rhodospirillales	206	66	26	19	90	8	14	8
other *Alphaproteobacteria*	81	27	20	16	36	2	6	2
Betaproteobacteria	41	30	37	33	11	3	7	3
Deltaproteobacteria								
SAR324	62	26	31	21	17	4	10	4
Bdellovibrionales Bacteriovoraceae	34	6	22	15	29	3	9	3
Desulfarculales Desulfarculacea	16	11	67	40	7	4	20	4
Desulfobacterales Desulfobacteraceae	1255	1074	906	772	130	46	116	46
Desulfobacterales Desulfobulbaceae	34	11	52	46	10	3	9	3
Desulfobacterales Nitrospinaceae	75	51	45	14	21	6	22	6
other *Desulfobacterales*	6	4	22	8	3	1	12	1
Desulfuromonadales Desulfuromonadaceae	60	0	3	0	5	0	3	0
Desulfuromonadales Geobacteraceae	16	5	10	4	10	2	6	2
other *Desulfuromonadales*	45	27	14	12	18	4	6	4
Desulfovibrionales	1	0	1	0	1	0	1	0
Myxococcales	59	21	75	39	35	4	34	4
Syntrophobacterales	32	24	87	77	9	1	7	1
other *Deltaproteobacteria*	241	109	155	92	122	31	76	31
Epsilonproteobacteria								
Campylobacterales Campylobacteraceae	159	1	2	1	25	1	2	1
Campylobacterales Helicobacteraceae	539	443	260	238	47	8	29	8
Nautiliales	5	0	1	0	3	0	1	0
other *Epsilonproteobacteria*	0	0	1	0	0	0	1	0
Gammaproteobacteria								
Acidithiobacillales Acidithiobacillaceae	265	241	525	486	28	15	40	15
Alteromonadales	57	33	45	43	20	6	8	6
Bd7-8	82	81	101	101	2	1	1	1
Chromatiales Chromatiaceae	47	39	20	17	11	5	8	5
Chromatiales Ectothiorhodospiraceae	7	3	9	2	4	2	6	2
Chromatiales Granulosicoccaceae	6	1	6	6	5	1	1	1
Chromatiales Nitrosococcus	1	1	4	4	1	1	1	1
Methylococcales	277	246	72	63	32	8	13	8
Oceanospirillales	57	20	40	35	25	4	9	4
other sulfur-oxidizing group	179	173	271	264	13	9	13	9
Thiotrichales Piscirickettsiaceae	97	57	68	54	21	6	13	6
other *Thiotrichales*	138	123	122	104	25	14	30	14
Xanthomonadales	189	124	119	103	58	19	34	19
other *Gammaproteobacteria*	301	166	415	312	115	25	98	25
Zetaproteobacteria	1	1	10	6	1	1	4	1
other *Proteobacteria*	52	23	70	58	21	5	17	5
Spirochaetes	160	103	57	24	30	7	25	7
Tenericutes	39	0	2	0	14	0	1	0
Verrucomicrobia	6	0	1	0	4	0	1	0
BRC1	19	2	14	2	16	2	13	2
OD1	3	0	12	0	2	0	12	0
OP3	27	8	44	9	18	4	29	4
OP9	2	2	32	5	1	1	3	1
OP11	0	0	6	0	0	0	5	0
SR1	56	0	4	0	35	0	3	0
TM7	1	0	1	0	1	0	1	0
WS3	60	30	112	51	29	15	56	15
TA06	24	13	59	12	14	6	38	6
TM6	38	14	34	7	23	3	28	3
other *Bacteria*	813	443	759	528	256	27	230	27
Total (*Bacteria*)	8030	4865	6972	4849	2335	549	1973	549

Table S5. Diversity and richness indexes of whole microbial community structures and each taxon/division at 5 and 25 cmbsf in methane seep sediment core 949C3. Tags with more than 98% identiity were assigned as same OTU.

	Tags		OTUs		Chao1		ACE		Shannon		Shannon eveness		Shimpson	
	5 cmbsf	25 cmbsf	5 cmbsf	25 cmbsf	5 cmbsf	25 cmbsf	5 cmbsf	25 cmbsf	5 cmbsf	25 cmbsf	5 cmbsf	25 cmbsf	5 cmbsf	25 cmbsf
Total	8709	7690	2473	2296	6558.0	6374.0	11117.8	11269.3	6.49	6.54	0.83	0.84	0.0063	0.0055
DHVEG6	74	162	47	120	130.3	405.0	151.5	436.8	3.45	4.65	0.90	0.97	0.0504	0.0054
MGI	535	81	50	12	65.5	(22.5)	71.7	(23.5)	2.22	(1.10)	0.57	(0.44)	0.2776	(0.5701)
Acidobacteria	311	290	161	125	426.4	200.9	607.3	339.4	4.64	4.44	0.91	0.92	0.0173	0.0149
Bacteroidetes	682	295	182	90	431.8	192.2	696.2	306.8	4.21	3.77	0.81	0.84	0.0388	0.0400
Chloroflexi	94	538	51	179	100.6	456.9	156.0	812.7	3.68	4.39	0.94	0.85	0.0229	0.0244
Planctomycetes	453	370	257	214	568.5	582.2	984.5	1125.6	5.21	5.06	0.94	0.94	0.0067	0.0068
Alphaproteobacteria	383	132	170	47	400.2	117.9	732.3	193.1	4.59	2.96	0.89	0.77	0.0172	0.1194
Gammaproteobacteria	1703	1817	362	278	880.0	616.9	1214.8	986.3	4.68	4.08	0.79	0.72	0.0226	0.0544
Deltaproteobacteria	1936	1490	415	331	1103.0	767.2	1649.2	1260.9	4.39	4.61	0.73	0.79	0.0443	0.0227
Epsilonproteobacteria	703	264	75	33	123.2	222.0	195.6	439.0	2.37	1.68	0.55	0.48	0.2091	0.3396

Tag population of the MGI at 25cmbsf was smaller than 100, and excluded from the discussion.
